# Integrative In Silico mRNA–miRNA Profiling of mTOR Pathway Dysregulation in High-Grade Serous Ovarian Carcinoma

**DOI:** 10.3390/cancers18050866

**Published:** 2026-03-07

**Authors:** Radwa Hablase, Cristina Sisu, Emmanouil Karteris, Jayanta Chatterjee

**Affiliations:** 1 College of Health, Medicine and Life Sciences, Brunel University of London, Uxbridge UB8 3PH, UK; cristina.sisu@brunel.ac.uk (C.S.); emmanouil.karteris@brunel.ac.uk (E.K.); jayanta.chatterjee1@nhs.net (J.C.); 2 Academic Department of Gynaecological Oncology, Royal Surrey NHS Foundation Trust Hospital, Guildford GU2 7XX, UK

**Keywords:** ovarian cancer, mTOR, TCGA, GTEx, miRNA

## Abstract

**Introduction and Background**: High-grade serous ovarian carcinoma (HGSOC) is notorious for its poor prognosis owing to its inherent biological aggressiveness and development of chemoresistance. The mechanistic target of rapamycin (mTOR) pathway is dysregulated in 55% of epithelial ovarian cancers, representing an appealing therapeutic target. To date, the clinical trials of mTOR inhibitors have shown modest response. In this study, we investigated the mTOR pathway in a clinical cohort of primary, chemo-naive, high-grade ovarian cancer samples, along with its regulatory post-transcriptional miRNA regulation. **Methodology**: We performed differential gene expression analysis on 100 HGSOC patients from TCGA and 80 healthy controls (i.e., normal ovarian tissue) from GTEx. The differentially expressed genes (DEGs) were overlaid onto the KEGG mTOR signalling pathway, followed by functional enrichment analysis. Next, we conducted differential miRNA expression analysis on the same cohort and identified regulatory miRNA–mTOR gene pairs involved in cancer pathogenesis. Finally, we constructed an interaction network and identified key hub genes and miRNAs with potential prognostic significance. **Results**: We identified 95 mTOR pathway genes that were significantly differentially expressed, involving upstream regulators, core components, and downstream effectors. Functional pathway analysis revealed a prominent shift toward mTORC1 activation, accompanied by paradoxical activation of autophagy. The let-7 miRNA family was identified as a key regulator of the mTOR pathway, potentially facilitating disease progression. RICTOR downregulation, a key component of the mTORC2 complex, appears to play a critical role in this histotype. In addition, FNIP1, a tumour suppressor gene implicated in mTOR dysregulation, was found to correlate with survival outcomes. **Conclusions**: We propose a model of dual activation of mTORC1 and autophagy in HGSOC as the metabolic rewiring enabling cancer progression under nutrient and cellular stress.

## Simple Summary

High-grade serous ovarian cancer (HGSOC) is the most common and aggressive type of ovarian cancer. It is often diagnosed at a late stage and eventually becomes resistant to standard chemotherapy. The mechanistic target of rapamycin (mTOR) is a key regulator of cellular functions including growth, survival, immune responses, and metabolism. To show how the mTOR pathway becomes dysregulated in HGSOC, we analysed gene expression data and microRNA patterns from both ovarian cancer patients and healthy individuals. We found that many of the genes involved in the mTOR pathway are unusually dysregulated. A group of regulatory molecules, the let-7 miRNAs, may allow this abnormal activity to continue. We also discovered a distinct pattern where one part of the pathway (mTORC1) is switched on while another (mTORC2) is switched off. These findings may help guide more effective, targeted treatments in the future targeting these pathways.

## 1. Introduction

Ovarian cancer is a lethal gynaecological malignancy [1]. The disease is characterised by significant heterogeneity in its histological subtypes, molecular profiles, and clinical behaviours [2]. High-grade serous ovarian carcinoma (HGSOC) is the commonest subtype and is notorious for its poor prognosis owing to late stage at diagnosis and inherent biological aggressiveness [3]. The mechanistic target of rapamycin (mTOR) is a serine/threonine protein kinase that belongs to the phosphatidylinositol 3-kinase-related kinase (PIKK) family. It functions as a central regulator of cellular homeostasis by balancing anabolic and catabolic processes in response to a variety of environmental cues, including growth factors, nutrient availability, energy status, oxygen tension, and genotoxic stress.

Activation of mTOR triggers anabolic programming, including lipid and protein biosynthesis, as well as angiogenesis, while repressing catabolic processes such as autophagy and apoptosis. Dysregulation of this pathway promotes tumorigenesis by sustaining growth and angiogenic signalling and suppressing cell death pathways [4].

The enzyme forms the catalytic subunit in two morphologically and functionally distinct complexes, namely mTORC1 and mTORC2 [5]. Both complexes share, in addition to mTOR, a scaffolding protein, mLST8 (Mammalian lethal with SEC13 protein 8), and a potential inhibitory regulator, Deptor (DEP domain-containing mTOR-interacting protein), but differ in their unique partners that define their substrate selectivity [6,7]. mTORC1 is characterised by the presence of Raptor (regulatory-associated protein of mTOR) and PRAS40 (proline-rich AKT substrate 40 kDa) [8]. In contrast, mTORC2 complexes with Rictor (Raptor-independent companion of mTOR), mSIN1 (stress-activated MAP kinase-interacting protein 1), and Protor-1 (Protein observed with RICTOR 1) [4].

Dysregulation of the mTOR pathway has been described in about 55% of epithelial ovarian cancers [9,10,11]. The molecular landscape of this dysregulation appears to be subtype-specific, with frequent mutations in key pathway regulators such as ARID1A (AT-rich interaction domain 1A), PIK3CA (Phosphatidylinositol-4,5-bisphosphate 3-kinase catalytic subunit alpha), and PTEN (Phosphatase and tensin homologue), which are rarely seen in HGSOC [12]. In HGSOC, TP53 (Tumour protein p53) mutations are almost always present, leading to widespread copy number alterations and genomic instability, which in turn drives hyperactivity of the mTOR pathway in this subtype [11,13,14].

To depict the dysregulation of the mTOR pathway in HGSOC, we carried out an integrative in silico analysis using transcriptomic and miRNA sequencing data from The Cancer Genome Atlas (TCGA) and Genotype-Tissue Expression (GTEx) consortia projects [15,16]. Differential expression analyses were conducted to identify significantly altered mTOR pathway genes and their regulatory miRNAs, as well as to construct the regulatory network. Finally, dimensionality reduction and survival analysis were performed to evaluate the diagnostic and prognostic significance of the identified hub genes within the mTOR signalling framework [17,18].

## 2. Materials and Methods

### 2.1. Dataset Selection

The high-grade serous ovarian cancer (HGSOC) transcriptomic data were obtained from the UCSC Xena Browser (https://xena.ucsc.edu/), the “TCGA TARGET GTEx” dataset. This Xena browser, Toil RNAseq recompute compendium, reprocessed unaligned RNA-seq reads from three public resources, The Cancer Genome Atlas, Genotype-Tissue Expression, and Therapeutically Applicable Research to Generate Effective Treatments (TARGET) projects, using the UCSC Computational Genomics Lab (CGL) RNA-seq pipeline. The data processing workflow was described previously [19].

Samples were filtered on the Xena browser based on the following phenotype parameters: Primary Site: Ovary; Sample Type: Normal Ovary (GTEx) and Primary Tumour (TCGA); Main Category: GTEx (GTEx ovary) and TCGA (ovarian serous cystadenocarcinoma). Those with history of neoadjuvant chemotherapy and unknown treatment status were excluded. Sample identifiers from both the TCGA and GTEx datasets were cross-referenced with their respective portals, and only samples with both mRNA and miRNA expression data on the TCGA and GTEx portals were included in the final analysis. (Sample IDs included in the study are detailed in Supplementary Table S1.)

### 2.2. Data Processing

For the transcriptomic data, the normalised gene expression matrix (RSEM norm_count) of the selected samples was downloaded from the Xena Browser, and no further processing was required. For the miRNA data, the GTEx gene expression read counts for small RNA-seq (not normalised) V10 were downloaded from the GTEx portal (https://www.gtexportal.org/home/downloads/adult-gtex/bulk_tissue_expression, accessed on 1 March 2026). The TCGA miRNA Isoform Expression Quantification files for the selected samples were downloaded from the GDC portal (https://portal.gdc.cancer.gov/). The details of the GTEx and TCGA preprocessing pipelines are available on their respective portals. We then used miRNA annotations from miRBase (v22.1), as implemented in Ensembl Genes release 115, accessed via the biomaRt package (v2.62.1), to identify and retain mature miRNAs from the two datasets.

Batch-effect correction was performed independently within each dataset, using the sequencing centre (SMCENTER) as the batch variable for GTEx samples and the plate ID for TCGA samples. The two datasets were then integrated, and an additional batch effect removal was performed using the sample dataset origin (GTEx versus TCGA) as the batch variable, to account for large-scale technical differences between the two resources. All batch corrections were performed on non-normalised read counts using the ComBat_seq function of the surrogate variable analysis (SVA v 3.54.0) package in R. This approach allows for removal of strong, systematic technical variation while preserving the underlying mean–variance relationship required for downstream count-based modelling. The resulting batch-adjusted count matrix was then used as input to DESeq2, which applies median-of-ratios normalisation and negative binomial modelling to estimate dispersion and test for differential expression. The level of batch effect on the data was assessed by principal component analysis (PCA) performed in R (version 4.4.1) using the plotPCA implemented in the DESeq2 package (v 1.46.0) (Supplementary Figures S1 and S2).

### 2.3. Differential Expression Analysis

The transcriptomic differential gene expression analysis was conducted using the LIMMA package in Bioconductor (https://www.bioconductor.org/) to estimate log fold changes and calculate adjusted *p*-values using the Benjamini–Hochberg correction method. Significantly dysregulated genes were defined as having an adjusted *p*-value < 0.05 and a log_2_FC (fold change) >0.5 or <−0.5 for upregulated and downregulated genes, respectively [20]. Differential expression analysis of miRNA counts was performed in R using the DESeq2 package (v 1.46.0) with integrated batch-corrected non-normalised read counts as input [21]. Differentially expressed miRNAs were defined based on an adjusted *p*-value of <0.05 and a log_2_FC (fold change) >0.5 or <−0.5 (Supplementary Table S2). The differential expression tools were optimised for distinct transcriptomic data structures and statistical distributions. This approach is consistent with established best practices, as limma performs robustly for normalised expression data, whereas DESeq2 is specifically designed for count-based small RNA sequencing data. The differentially expressed genes and miRNA were visualised using the ggplot2 (v 3.5.2) R package.

### 2.4. Transcriptomic Analysis

The KEGGREST (v 1.46.0) package in R was used to retrieve the list of genes involved in the mTOR signalling pathway (hsa04150) from the Kyoto Encyclopedia of Genes and Genomes (KEGG) database (release 117.0) [22,23]. Differentially expressed mTOR pathway genes in our dataset were filtered and visualised using the pheatmap (v 1.0.12) R package. To provide pathway-level biological context, differentially expressed mTOR pathway genes were overlaid on the KEGG mTOR pathway using the Pathview (v 1.46.0) R package [24]. Functional enrichment analysis was then performed using the gprofiler2 (v 0.2.3) R package.

### 2.5. Prediction and Integration of miRNA–mRNA Regulatory Interactions

Predicted gene targets of differentially expressed miRNAs were obtained using the miRDB database through the multiMiR (v 1.28.0) package in R [25,26]. Predicted targets were then intersected with the differentially expressed mTOR pathway genes identified in the mRNA analysis to generate a set of mTOR pathway miRNA–mRNA pairs potentially dysregulated in HGSOC, and only those experimentally validated were used to construct the network analysis (Supplementary Table S3).

### 2.6. Network Construction and Topological Analysis of mTOR Pathway miRNA–mRNA Interactions

Network analysis of mTOR pathway miRNA–mRNA interactions were performed using Cytoscape software (version 3.10.3), developed by the Cytoscape Consortium (Cytoscape Consortium, San Diego, CA, USA) [27] (Supplementary Table S3). Hub genes and miRNAs were identified through network-based centrality analysis combined with biological filtering. A directed miRNA–mRNA regulatory network was constructed using experimentally validated interactions exhibiting inverse expression patterns. Degree centrality, defined as the number of regulatory edges per node, was calculated. Nodes with high degree centrality, indicating regulation by multiple miRNAs or targeting of multiple mTOR pathway genes, were considered candidate hubs. To identify cancer-relevant hubs, the network was further filtered to include only miRNAs listed in the KEGG MicroRNAs in Cancer pathway (hsa05206) and genes belonging to the mTOR signalling pathway.

### 2.7. Survival Analysis

Kaplan–Meier analyses of overall survival (OS) were performed using the survival (v 3.8-3) package in R for each hub gene identified in the cancer regulatory network. Patients were stratified into high- and low-expression groups based on the median expression values, and survival curves were compared using the log-rank test.

### 2.8. Dimensionality Reduction of Hub Gene Expression Analysis

T-distributed stochastic neighbor embedding (T-SNE) analysis was performed using scaled expression values of the hub genes to visualise sample stratification between TCGA tumour and GTEx normal tissues, using the Rtsne (v 0.17) package of R.

**Figure 1:**
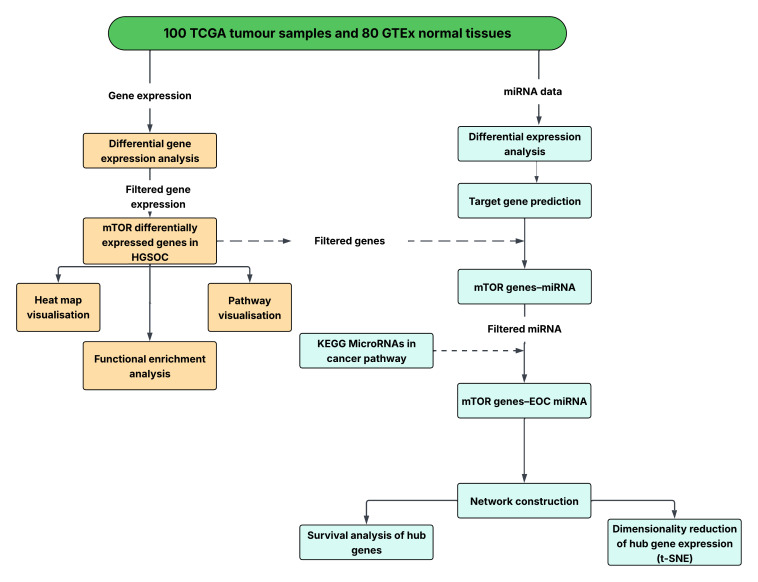
The study workflow. Differential gene and miRNA expression analysis was performed on 100 HGSOC and 80 normal ovarian tissues. Differentially expressed genes (DEGs) were filtered for those involved in the KEGG mTOR signalling pathway, and pathway-level visualisation was performed. Differentially expressed miRNA target gene prediction was performed. The KEGG “MicroRNAs in Cancer” pathway was used to filter miRNA–mTOR gene pairs dysregulated in high-grade serous ovarian cancer. A regulatory network representing mTOR-specific miRNA interactions in HGSOC was constructed. Survival analysis and dimensionality reduction of hub nodes were performed.

An overview of the present study workflow is shown in Figure 1.

## 3. Results

### 3.1. Transcriptomic Profiling of mTOR Pathway in High-Grade Serous Ovarian Cancer: A Pathway-Level Analysis

The study cohort included 100 HGSOC and 80 normal ovarian samples from the TCGA and GTEx databases, respectively. The cancer samples were primary ovarian tissues and high-grade (G2 and G3), with no prior history of neoadjuvant chemotherapy. The UCSC Xena Browser Toil RNA-seq normalised read counts were retrieved, and differential gene expression analysis was performed using the LIMMA package in R (v3.62.2).

**Figure 2:**
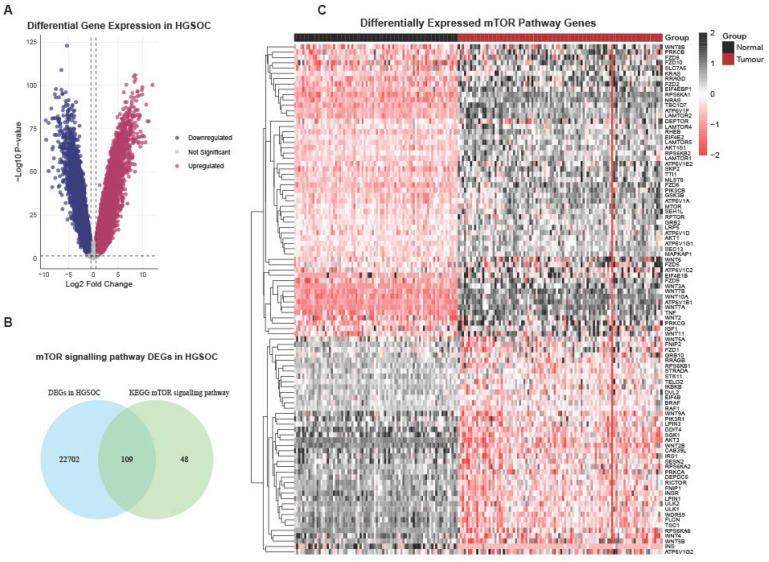
(**A**) Volcano plot showing 58,581 expressed genes in HGSOC samples compared to normal ovarian tissue. The significance threshold applied was a *p*-adjusted value < 0.05 and |log_2_FC| > 0.5. The blue dots represent statistically significant downregulated genes, the red dots represent statistically significantly upregulated genes, and the grey dots represent differentially expressed genes that did not pass the significance thresholds. The green horizontal dashed line represents adj. *p* < 0.05. (**B**) Venn diagram highlighting the intersection between the 22,811 significantly differentially expressed genes (DEGs) in high-grade serous ovarian cancer (HGSOC) and the 157 genes included in the KEGG mTOR signalling pathway. A total of 96 mTOR pathway genes were significantly differentially expressed in HGSOC. (**C**). Heat map of differentially expressed mTOR pathway genes in HGSOC showing expression levels across tumour (red) and normal ovarian tissue samples (black) (top bar). Rows represent genes; columns represent individual samples.

The differential gene expression analysis revealed that, of the 58,581 genes expressed in HGSOC and normal ovarian tissues, 22,811 were significantly differentially expressed (DEGs) using the cut-off values of *p*-adjusted < 0.05 and |log_2_FC| > 0.5. A total of 12,802 genes were upregulated and 10,009 were downregulated (Figure 2A). Subsequently, the 22,811 genes were intersected with the KEGG mTOR pathway, yielding 96 pathway-specific genes dysregulated in HGSOC (Figure 2B and Supplementary Table S4).

A heat map of the 96 mTOR pathway genes was subsequently constructed, revealing clear clustering between tumour and normal ovarian tissue (Figure 2C). A total of 55 genes were upregulated and 41 downregulated (Supplementary Figure S3). The core mTORC1/2 complex genes that met the significance thresholds included mTOR, Deptor, Raptor, Rictor, mLST8, AKT1S1, and MAPKAP1; all but Rictor were upregulated. Despite the modest fold changes of the mTORC1/2 core complex genes, several upstream effectors displayed significant upregulation. By contrast, DEPDC5 (DEP domain-containing protein 5) and TSC1 (Tuberous sclerosis complex 1) were among the downregulated genes. Collectively, these findings suggest a transcriptional reprogramming of the mTOR signalling pathway in HGSOC, characterised by concurrent activation of upstream effectors and attenuation of selective inhibitory regulators.

**Figure 3:**
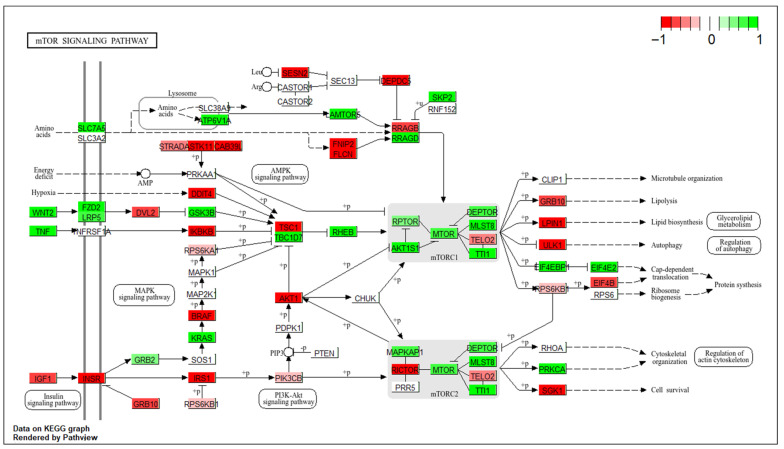
Pathway-level mTOR signalling dysregulation in HGSOC. The diagram illustrates the KEGG mTOR signalling pathway (hsa04150), overlaid with mTOR differentially expressed genes from our study cohort. Genes are colour-coded based on log_2_FC values: green indicates upregulated genes, red indicates downregulated genes, and white denotes genes with no significant differential expression. The colour bar on the top right corner displays the colour intensity scale from −1 to +1, corresponding to the full range of observed log fold change values.

To better understand the functional interactions, we overlaid mTOR DEGs onto the KEGG mTOR pathway (hsa04150) (Figure 3). To analyse the transcriptomic dysregulation of the mTOR pathway in HGSOC, we first examined the canonical pathway, then explored mTOR interactions with various other pathways, and finally analysed the amino acid upstream effectors.

The IGF-1 (Insulin-like growth factor 1) signalling in HGSOC appears to be geared toward the RAS–RAF–MEK–ERK (Rat sarcoma–rapidly accelerated fibrosarcoma–mitogen-activated protein kinase–extracellular signal-regulated kinase) pathway, driven by IGF-1 upregulation, IRS1 (insulin receptor substrate 1) downregulation, and GRB2 (receptor-bound protein 2) upregulation. PI3K (Phosphatidylinositol-3-kinase) activation appears less dependent on insulin and IGF-1, with increased PIK3CB (p110β) (phosphatidylinositol-4,5-bisphosphate 3-kinase catalytic subunit beta) and reduced PIK3R1 (p85α) (phosphatidylinositol-3-kinase regulatory subunit 1) weakening negative regulation and allowing enhanced catalytic activity. Akt (Protein kinase B) signalling was also altered, with AKT1 (AKT serine/threonine kinase 1) upregulated, AKT3 (AKT serine/threonine kinase 3) downregulated, and reduced Rictor expression shifting activity away from mTORC2 toward dominant mTORC1 signalling. Multiple upstream inputs, including downregulation of TSC1, altered RSK (ribosomal S6 kinase) isoforms, and reduced REDD1 (DNA damage responses 1), can collectively suppress the TSC1/TSC2 complex, sustaining mTORC1 activation. Energy-stress inhibition of mTORC1 was compromised by downregulation of CAB39L (calcium-binding protein 39-like), STRADA (STE20-related adaptor protein alpha), and LKB1 (liver kinase B1), impairing AMPK (AMP-activated protein kinase) activation. Amino acid-sensing mechanisms were strongly activated through upregulation of SLC7A5 (family 7 member 5), Ragulator, and RagD (Ras-related GTP-binding protein D), alongside downregulation of Sestrins, DEPDC5, and FLCN/FNIP (folliculin/folliculin-interacting protein complex). Collectively, these transcriptomic changes support a model of persistent mTORC1 activation coupled with increased autophagy, enabling metabolic adaptation and tumour progression in HGSOC.

**Figure 4:**
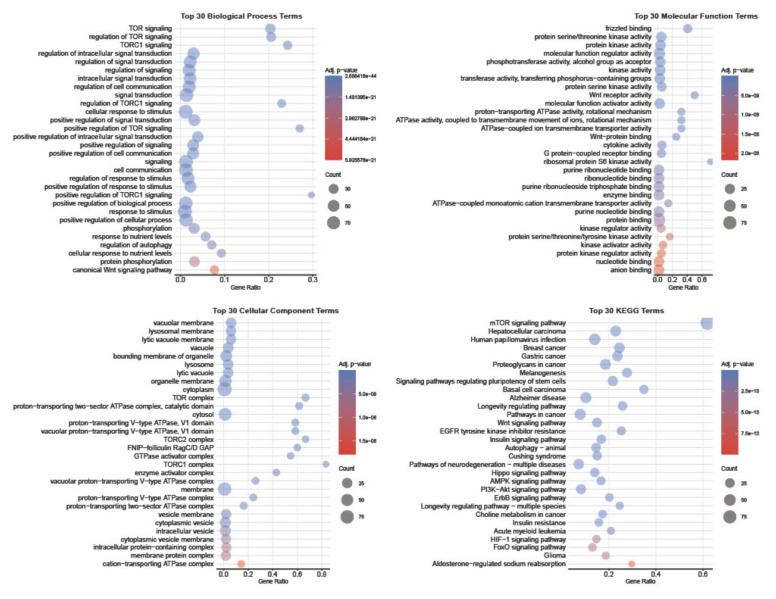
Functional enrichment analysis of differentially expressed mTOR pathway genes in HGSOC. Gene ontology and KEGG pathway enrichment were performed. The top 30 significantly enriched terms are shown for biological process, molecular function, cellular component, and KEGG pathways.

Functional enrichment analysis was then performed to systematically characterise the broader biological roles and molecular interactions of the differentially expressed mTOR pathway genes in HGSOC. The enrichment results were summarised across gene ontology (GO) domains, biological process (BP), molecular function (MF), and cellular component (CC), as well as KEGG pathways (Figure 4).

Among the top enriched gene ontology biological process terms, and in addition to the expected significantly enriched TOR-related signalling, were processes associated with “regulation of autophagy” and “canonical Wnt signalling pathway”. The molecular function terms were dominated by kinase-related activities, including protein serine/threonine kinase activity, together with frizzled binding and Wnt-protein binding. At the cellular component level, the enriched terms were primarily associated with lysosomal and vacuolar membranes and the TOR complex. KEGG pathway enrichment reinforced these findings, identifying overrepresentation of the mTOR signalling pathway, PI3K–Akt, AMPK, Wnt, and multiple cancer-related pathways.

### 3.2. The Regulatory miRNA Network of mTOR Pathway Genes in High-Grade Serous Ovarian Cancer

For consistency with the mRNA analysis, we used the same patient cohorts to examine the miRNA regulatory network. Raw miRNA count reads for these samples were retrieved from the GDC TCGA and GTEx databases, matched by the sample IDs.

**Figure 5:**
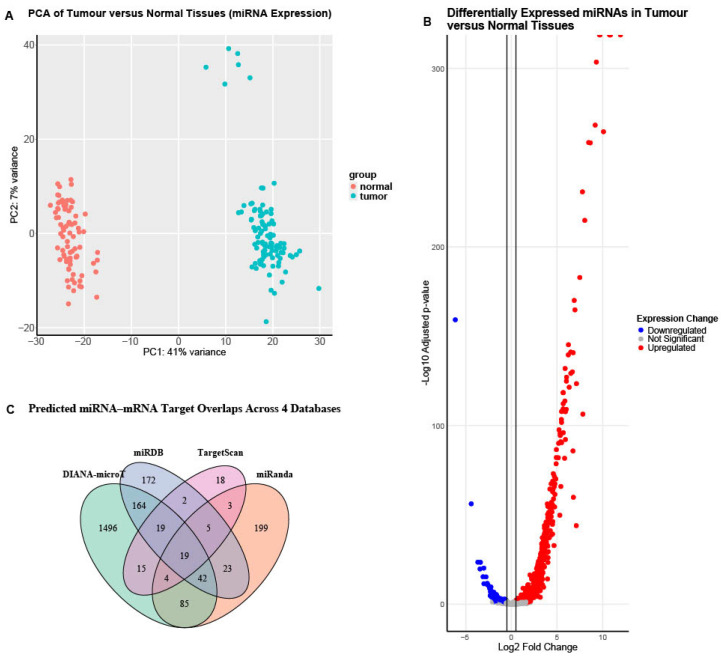
(**A**) Principal component analysis of miRNA expression after batch correction and normalisation. PCA plot showing the separation of HGSOC tumour and normal ovarian tissues based on normalised and batch-corrected miRNA expression profiles. The clear distinction along the first principal component (PC1, 41% variance) indicates substantial expression differences between tumour and normal groups, reflecting distinct miRNA regulatory landscapes in HGSOC. (**B**) Volcano plot of differentially expressed miRNAs in HGSOC tumour versus normal ovarian tissues. The significance thresholds applied were an adjusted *p*-value < 0.05 and a |log_2_FC| > 0.5. Red dots represent significantly upregulated miRNAs, blue dots represent significantly downregulated miRNAs, and grey dots represent miRNAs that did not pass the significance thresholds. The green horizontal dashed line indicates the adjusted *p*-value cutoff of 0.05. (**C**). Venn diagram illustrating the overlap of predicted miRNA–mTOR gene interactions obtained from DIANA-microT, miRanda, miRDB, and TargetScan. Predictions were based on the 621 significantly differentially expressed miRNAs and the 96 differentially expressed mTOR pathway genes identified in our HGSOC cohort. Only interactions predicted in two or more databases were considered reliable, resulting in a total of 381 high-confidence miRNA–mTOR gene pairs retained for further analysis.

For quality assessment, principal component analysis (PCA) was performed before and after batch correction, and boxplots were generated before and after normalisation (Supplementary Figures S1 and S2). A final PCA plot was constructed using the batch-corrected and normalised read counts (Figure 5A).

Subsequently, differential miRNA expression analysis was performed using DESeq2. A total of 1333 miRNAs were differentially expressed between tumour (HGSOC) and normal tissues, with 621 meeting the significance threshold (adjusted *p*-value < 0.05 and lo_g2_FC > 0.5). Of these, 546 were upregulated, and 75 were downregulated (Figure 5B) (Supplementary Table S2).

Predicted gene targets for the significantly differentially expressed miRNAs were identified. miRNA–mTOR gene interactions were retrieved from four major databases: TargetScan, miRDB, miRanda, and DIANA-microT [24,27,28,29]. To minimise false-positive predictions, only interactions predicted in two or more databases were retained, resulting in 381 high-confidence miRNA–mTOR gene pairs (Figure 5C).

**Figure 6:**
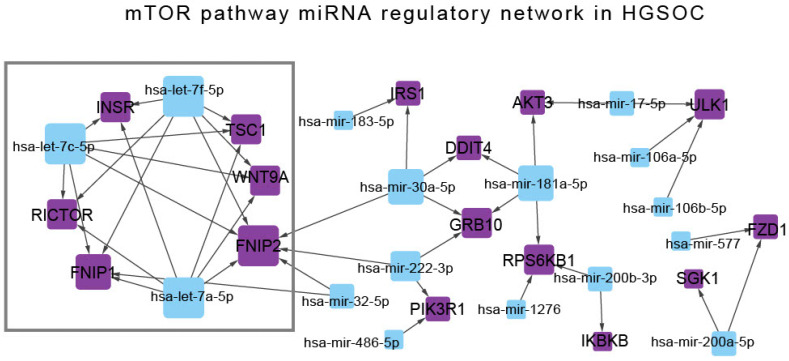
mTOR–miRNA regulatory network in high-grade serous ovarian carcinoma (HGSOC). The network illustrates the interactions between significantly differentially expressed mTOR pathway genes (purple nodes) and their experimentally validated regulatory miRNAs (blue nodes). Each edge represents an inverse expression relationship between an miRNA and its mTOR target gene, as identified from multiMiR. The node size corresponds to the number of edges to or from the node. The resulting network highlights 43 regulatory pairs involving 16 mTOR pathway genes and 16 miRNAs.

These pairs were subsequently evaluated for experimental validation using multiMiR. In multiMiR, “validated” interactions refer to miRNA–target gene pairs that have been experimentally confirmed in prior studies using methods such as luciferase reporter assays, Western blotting, qPCR (Quantitative Polymerase Chain Reaction), and immunoprecipitation [26]. That yielded 64 validated miRNA–mTOR gene interactions. These pairs comprised differentially expressed miRNAs and mTOR pathway DEGs identified in our HGSOC cohort. Based on expression profiles, only pairs demonstrating inverse expression patterns (i.e., upregulated miRNAs with downregulated target genes or vice versa) were considered biologically relevant. A total of 43 such regulatory pairs were retained and used to construct the mTOR–miRNA regulatory network in HGSOC (Figure 6) (Supplementary Table S3).

To identify hub nodes within the network, degree centrality was calculated, and a score of three or greater was used to define hub nodes. Among the miRNAs, hsa-let-7f-5p, hsa-let-7c-5p, and hsa-let-7a-5p had the highest degree centrality, followed by hsa-miR-181a-5p, hsa-miR-30a-5p and hsa-miR-222-3p (Table 1).

Among the mTOR pathway genes, FNIP2 (folliculin-interacting protein 2) had the highest degree centrality, followed by FNIP1 (folliculin-interacting protein 1). GRB10 (Growth factor receptor-bound protein 10), RPS6KB1 (ribosomal protein S6 kinase B1), ULK1 (unc-51-like autophagy-activating kinase 1), WNT9A (wingless-type MMTV integration site family member 9A), TSC1, RICTOR, and INSR (insulin receptor) each interacted with three miRNAs and were therefore also classified as hub genes (Table 1).

The three most highly connected miRNAs identified, hsa-let-7f-5p, hsa-let-7c-5p, and hsa-let-7a-5p, were cross-referenced against the KEGG MicroRNAs in Cancer pathway (hsa05206) to confirm their relevance to cancer pathogenesis. All three miRNAs were annotated within this pathway. These miRNAs were found to co-target six mTOR pathway-related genes: FNIP1, FNIP2, INSR, RICTOR, TSC1, and WNT9A. The convergence of these cancer-associated miRNAs on a shared set of mTOR-related genes highlights their potential relevance to the molecular pathogenesis of high-grade serous ovarian cancer.

**Figure 7:**
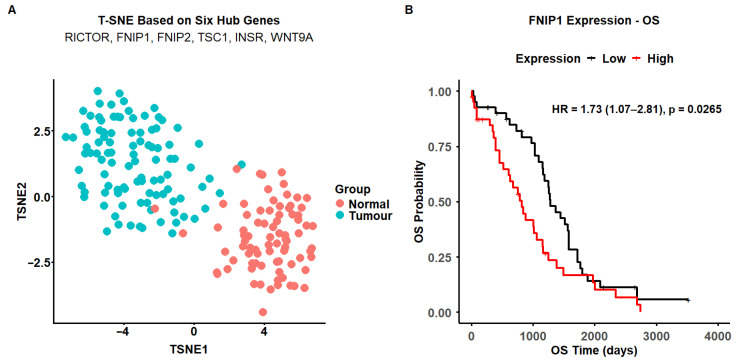
(**A**) T-distributed stochastic neighbor embedding (T-SNE) analysis was performed using scaled expression values of six hub genes (RICTOR, FNIP1, FNIP2, TSC1, INSR, and WNT9A). The plot illustrates clear stratification between TCGA tumour samples (blue) and GTEx normal tissues (red), indicating distinct expression patterns of these hub genes between tumour and normal groups. T-SNE was used as an exploratory visualisation approach. (**B**) Kaplan–Meier analysis of FNIP1 expression and overall survival (OS) in HGSOC. Patients were stratified into high- and low-FNIP1 expression groups based on the median transcript level. Kaplan–Meier curves show that higher FNIP1 expression is associated with significantly improved overall survival (log-rank *p* = 0.024). The Cox proportional hazards model yielded a hazard ratio (HR) = 1.73 (95% CI 1.07–2.81; *p* = 0.0265), indicating that patients with low FNIP1 expression have a 1.7-fold greater risk of death compared with those with high expression. Shaded areas represent 95% confidence intervals.

To assess the network hub genes’ potential to inform the disease diagnostic, we performed an exploratory dimensionality reduction analysis (T-SNE) using the gene expression data, which showed clear stratification between TCGA and GTEx samples, suggesting that the identified hub genes may collectively be regarded as a diagnostic marker panel (mean silhouette score = 0.60) (Figure 7A).

Next, we assessed the prognostic relevance of these genes in HGSOC, evaluating the overall survival (OS) across all six genes in our cohort (Supplementary Figure S4). Among these genes, FNIP1 demonstrated a significant association with improved overall survival (Figure 7B).

## 4. Discussion

mTOR signalling pathway hyperactivation has been reported in 70% of ovarian cancer cases and is thought to drive tumour growth, metabolic adaptation, and resistance to therapy [28]. Despite the measurable pathway activation, numerous first-generation mTOR inhibitors (rapalogues) have been evaluated clinically, yet their impact has been modest, an outcome often attributed to complex feedback loops and compensatory signalling mechanisms that blunt the therapeutic efficacy [29]. Furthermore, epithelial ovarian cancers (EOCs) are further classified into at least five different histological subtypes, and the molecular mechanisms underlying the pathway dysregulations appear to be subtype-specific [11].

Here, we profiled the entire mTOR pathway in primary, chemotherapy-naïve HGSOC samples in silico, capturing the full spectrum of transcriptional alterations across its upstream, core, and, to a lesser extent, downstream effectors in a subset of patients from the TCGA and GTEx databases. We limited the sample selection to those where both transcriptional and miRNA data were available. We then integrated these observed transcriptional changes with the miRNA regulatory network obtained from the same samples. By doing so, we moved beyond the traditional linear view of canonical versus non-canonical signalling. This pathway-level integration enabled us to identify patterns of coordinated or co-occurring transcriptional events that could potentially be functionally linked through shared miRNA regulation. The study offers a new perspective on how mTOR pathway hyperactivity is sustained in HGSOC and identifies potential regulatory nodes that may have diagnostic and prognostic value.

In HGSOC, we noted less pathway reliance on IGF-1 activation, demonstrated by transcriptional downregulation of IGF-1. This aligns with the work done by King et al., demonstrating that IGF-1 and IGF1R (Insulin-like growth factor 1 receptor) were more overexpressed in low-grade serous than in high-grade serous disease and that low-grade cell lines were more responsive to IGF-1 stimulation and IGF-1R inhibition than high-grade cell lines [30].

We also noted transcriptional downregulation of RICTOR, a key component of mTORC2, shifting the pathway towards more mTORC1 prominence, consistent with previous reports demonstrating that ovarian cancer cells predominantly rely on mTORC1-mediated signalling for growth and metabolic adaptation [31]. TSC1 downregulation further removes the brakes from mTORC1, supporting alternate pathway activation independent of insulin and insulin growth factor upstream signalling [32].

The Wnt (Wingless-related integration site) signalling pathway was among the enriched gene ontology terms in our cohort, perhaps indicating intricate crosstalk between the two pathways. However, we noticed transcriptional downregulation of DVL3 (Dishevelled segment polarity protein 3) and upregulation of GSK3B (Glycogen synthase kinase 3 beta). The functional interpretation of this pattern is difficult to depict, as the current literature supports both pro-oncogenic and tumour-suppressive effects with reported variations across samples of the same histotype [33]. This distinct transcriptomic pattern highlights a potential node of pathway crosstalk between the mTOR and Wnt networks. This observation provides a compelling foundation for future functional studies to delineate the precise mechanistic implications of GSK3B dysregulation in HGSOC.

In our cohort, amino acid-driven activation of mTORC1 displayed complex interaction patterns. For example, upregulation of the amino acid transporter SLC7A5 (LAT1), which mediates leucine uptake and stimulates Ragulator–Rag GTPase activation, has been widely shown to enhance mTORC1 signalling and promote tumour growth in ovarian and other cancers [34]. Similarly, activation of the V-ATPase–Ragulator complex (Vacuolar H+-ATPase–Ragulator complex) provides a lysosomal scaffold required for amino acid sensing and mTORC1 recruitment to the lysosomal surface [35,36]. In contrast, the downregulation of DEPDC5, a key component of the GATOR1 (GAP activity toward Rags complex 1) complex that inhibits Rag GTPases under amino acid deprivation, relieves this inhibitory brake and further favours sustained mTORC1 activation [37]. The FLCN–FNIP complex has been reported to be downregulated or functionally inactivated in multiple cancers [38,39]. Mechanistically, loss of FNIP1 or FNIP2 disrupts the FLCN–FNIP–AMPK axis and releases transcription factors TFEB (Transcription factor EB) and TFE3 (Transcription factor E3) from lysosomal tethering, allowing for their nuclear translocation and subsequent activation of genes involved in autophagy and lysosomal biogenesis [40]. This transcriptional programme includes upregulation of RAG-D, which enhances Rag GTPase-mediated mTORC1 activation at the lysosomal surface [41]. Consequently, FLCN/FNIP downregulation paradoxically results in both mTORC1 hyperactivation and increased autophagy, suggesting a cancer-promoting feedback mechanism whereby autophagy sustains intracellular nutrient levels to support continued growth and survival under metabolic stress [42]. However, it is critical to acknowledge that the canonical suppression of autophagy by mTORC1 occurs predominantly post-translationally, primarily through the inhibitory phosphorylation of the ULK1 complex [43]. Furthermore, transcriptional upregulation of autophagy-related genes does not inherently reflect increased or active autophagic flux, as steady-state mRNA levels do not account for protein translation efficiency, post-translational modifications, or lysosomal degradation dynamics [44]. Therefore, while our in silico findings highlight an intriguing transcriptional dynamic regarding simultaneous mTORC1 and autophagy activation, future in vitro and in vivo studies utilising targeted proteomics, phosphoproteomics, and dynamic autophagic flux assays (e.g., LC3 turnover) are essential to definitively determine whether this simultaneous transcriptional upregulation translates into functional, concurrent pathway activity at the protein level in HGSOC.

As mentioned, miRNAs are small non-coding RNAs, typically 21–25 nucleotides in length, that regulate gene expression at the post-transcriptional level by promoting mRNA degradation or inhibiting translation [45]. Among them, the let-7 family represents one of the most evolutionarily conserved and extensively studied groups of miRNAs [46]. Traditionally regarded as tumour suppressors, let-7 members are frequently downregulated in human cancers, where their loss contributes to enhanced oncogene expression and dedifferentiation [47]. However, emerging evidence suggests that the functional role of let-7 miRNAs is context-dependent; in certain tumour microenvironments or stress-adapted states, let-7 upregulation may facilitate metabolic adaptation, autophagy, or dormancy, reflecting a more complex and dynamic contribution to tumour progression than previously appreciated [48].

In line with our results, previous experimental work in gastric cancer demonstrated that miR-let-7a directly targets RICTOR, a key component of the mTORC2 complex, thereby enhancing autophagy. Upregulation of miR-let-7a was shown to increase autophagic flux, while its suppression reduced autophagy, confirming a regulatory link between let-7a expression, RICTOR downregulation, and autophagy activation [48]. Similarly, in our study, we observed upregulation of miR-let-7a accompanied by downregulation of RICTOR, suggesting that a comparable let-7a–RICTOR–mTOR axis may contribute to autophagy-mediated adaptation in high-grade serous ovarian carcinoma.

Indeed, the let-7 microRNA family plays an important role in metabolic reprogramming in both physiological and pathological states, influencing cellular energy balance, mitochondrial function, and nutrient sensing. In our study, we demonstrated a clear interaction between let-7 miRNAs and the mTOR metabolic signalling network in HGSOC, highlighting a potential post-transcriptional mechanism through which let-7 contributes to the metabolic plasticity and survival of tumour cells under stress conditions [49].

Collectively, evidence across several cancer models suggests that the let-7 family functions as a context-dependent regulator of autophagy. In lung cancer, let-7 induces autophagy by targeting IGF1R and disrupting the BCL2L1/BCL2/PI3K (BCL2-like 1/B-cell lymphoma 2/phosphoinositide 3-kinase) complex. In gastric cancer, let-7a suppresses the mTORC2 component RICTOR, thereby activating autophagy. Similarly, in human placental trophoblasts, let-7b promotes autophagy through a TGFBR1/ERK/IL-6/TNF-α (Transforming growth factor beta receptor 1/extracellular signal-regulated kinase/interleukin 6/tumour necrosis factor alpha) cascade, while in glioma, let-7a/d/f downregulate STAT3 (Signal transducer and activator of transcription 3), inducing both apoptosis and autophagy [50]. Taken together, these findings indicate that let-7 miRNAs are versatile modulators of autophagic fluxes, exerting pro- or anti-autophagic effects depending on the cellular and metabolic context. In our study, the upregulation of let-7a, let-7c, and let-7f in HGSOC, coupled with downregulation of RICTOR and FNIP1, supports a model in which let-7-mediated autophagy activation may contribute to metabolic adaptation and survival under nutrient and metabolic stress.

Our findings are consistent with previous mechanistic studies in epithelial ovarian cancer showing that let-7 miRNAs modulate AKT–mTOR signalling by altering phosphorylation dynamics at AKT1. Boyd et al. demonstrated that let-7a overexpression reduces S473 phosphorylation (a RICTOR/mTORC2-dependent site) while increasing T308 phosphorylation via PDK1 (3-phosphoinositide-dependent protein kinase 1), thereby shifting signalling balance toward mTORC1 activation and autophagy. This aligns with our observation of upregulated let-7a/c/f and downregulation of RICTOR in HGSOC, suggesting that let-7-mediated reprogramming of AKT phosphorylation may underpin the autophagic and metabolic adaptations characteristic of HGSOC [51]. In addition, according to the Human Protein Atlas, FNIP1 has a potential prognostic value in ovarian cancer, with higher expression associated with worse outcome [52]. To date, however, we are not aware of a dedicated study evaluating FNIP1 as a prognostic marker specifically in HGSOC [52]. A key limitation of this study is the use of bulk ovarian tissue from GTEx as the normal control, which comprises heterogeneous cell populations including stromal and follicular components. As high-grade serous ovarian cancer is now widely recognised to originate predominantly from fallopian tube epithelium, some observed differential gene expression may reflect tissue composition differences rather than cancer-specific dysregulation alone. Ideally, fallopian tube epithelial tissue would represent the most biologically appropriate comparator; however, such datasets with comparable scale and sequencing consistency are currently limited. Therefore, our findings should be interpreted within this biological context, while recognising the established utility of GTEx as a reference in large-scale transcriptomic analyses [19,53].

## 5. Conclusions

This study provides an in silico integrated transcriptomic and post-transcriptional overview of the mTOR signalling pathway in HGSOC. We identified three members of the let-7 family—let-7a-5p, let-7c-5p, and let-7f-5p—as central regulatory nodes within the mTOR signalling network in this gynaecological malignancy. These miRNAs co-targeted six key mTOR-related genes (FNIP1, FNIP2, INSR, RICTOR, TSC1, and WNT9A), linking them to metabolic and autophagic regulation. The dual activation of mTORC1 and autophagy suggests a cancer-promoting adaptation that enables tumour cells to maintain intracellular nutrient balance and survive metabolic stress. The model proposed represents an under-researched area, and further in vitro and in vivo investigations are warranted to validate the regulatory relationships identified and to elucidate their functional significance within the mTOR signalling and metabolic adaptation framework in HGSOC.

## Figures and Tables

**Table 1:** mTOR pathway hub nodes in HGSOC.

Node	Node Type	Degree Centrality
hsa-let-7a-5p	miRNA	6
hsa-let-7c-5p	miRNA	6
hsa-let-7f-5p	miRNA	6
hsa-mir-181a-5p	miRNA	4
hsa-mir-30a-5p	miRNA	4
hsa-mir-222-3p	miRNA	3
FNIP2	mRNA	6
FNIP1	mRNA	4
GRb10	mRNA	3
INSR	mRNA	3
RICTOR	mRNA	3
RBS6KB1	mRNA	3
TSC1	mRNA	3
ULK1	mRNA	3
WNT9A	mRNA	3

## Data Availability

The original contributions presented in this study are included in the article/Supplementary Material. Further inquiries can be directed to the corresponding author.

## Supplementary Materials

The following supporting information can be downloaded at: https://www.mdpi.com/article/10.3390/cancers18050866/s1. Figure S1. Quality assessment of batch correction by principal component analysis. Figure S2. Global miRNA expression before and after DESeq2 normalisation. Figure S3. Differential expression of mTOR pathway genes in high-grade serous ovarian cancer. Figure S4. Kaplan–Meier survival analysis of six hub genes in high-grade serous ovarian cancer. Table S1. Sample identifiers and source databases of the study cohort. Table S2. miRNA differential expression analysis. Table S3. Predicted miRNA targets, validation status, and mTOR-specific cancer-related miRNA–mRNA interactions, degree centrality. Table S4. mRNA differential expression analysis.
