# Supplementary material for: Integrative In Silico mRNA–miRNA Profiling of mTOR Pathway Dysregulation in High-Grade Serous Ovarian Carcinoma
Source: Cancers (Basel). 2026 Mar 7;18(5):866. doi: 10.3390/cancers18050866 (PMC12984994; doi:10.3390/cancers18050866)
Supplement: Supplementary file 1 [file cancers-18-00866-s001.zip › Supplementary.pdf]

Supplementary Figures and Tables.

Supplementary Figure S1. Quality assessment of batch correction by principal component analysis.

Principal component analysis (PCA) of miRNA-seq expression profiles from normal ovarian (GTEx) and high-grade serous ovarian cancer (HGSOC; TCGA) samples, before and after ComBat-seq batch correction. Before correction, samples clustered predominantly by dataset of origin, indicating strong batch effects. After ComBat-seq correction, clustering was primarily driven by biological condition (normal versus tumour), demonstrating effective removal of batch-related variation while preserving the biological signal.

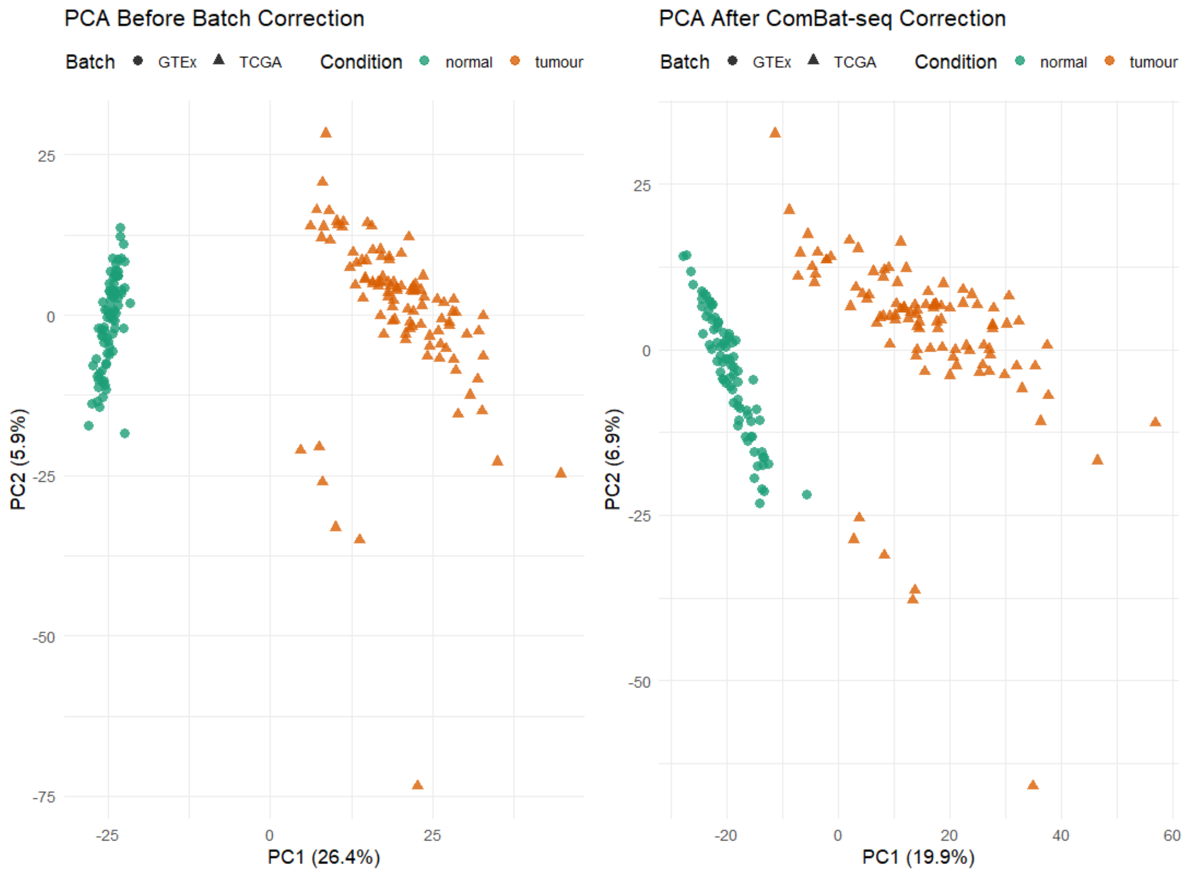

Supplementary Figure S2. Global miRNA expression before and after DESeq2 normalisation.

Boxplots show the distribution of log_2-transformed miRNA expression levels across samples before and after DESeq2 size-factor normalisation. Lowly expressed genes (counts ≤1 in ≥90% of samples) were excluded to improve visual clarity. Normalisation aligned median expression levels across samples, indicating effective correction for library size variation.

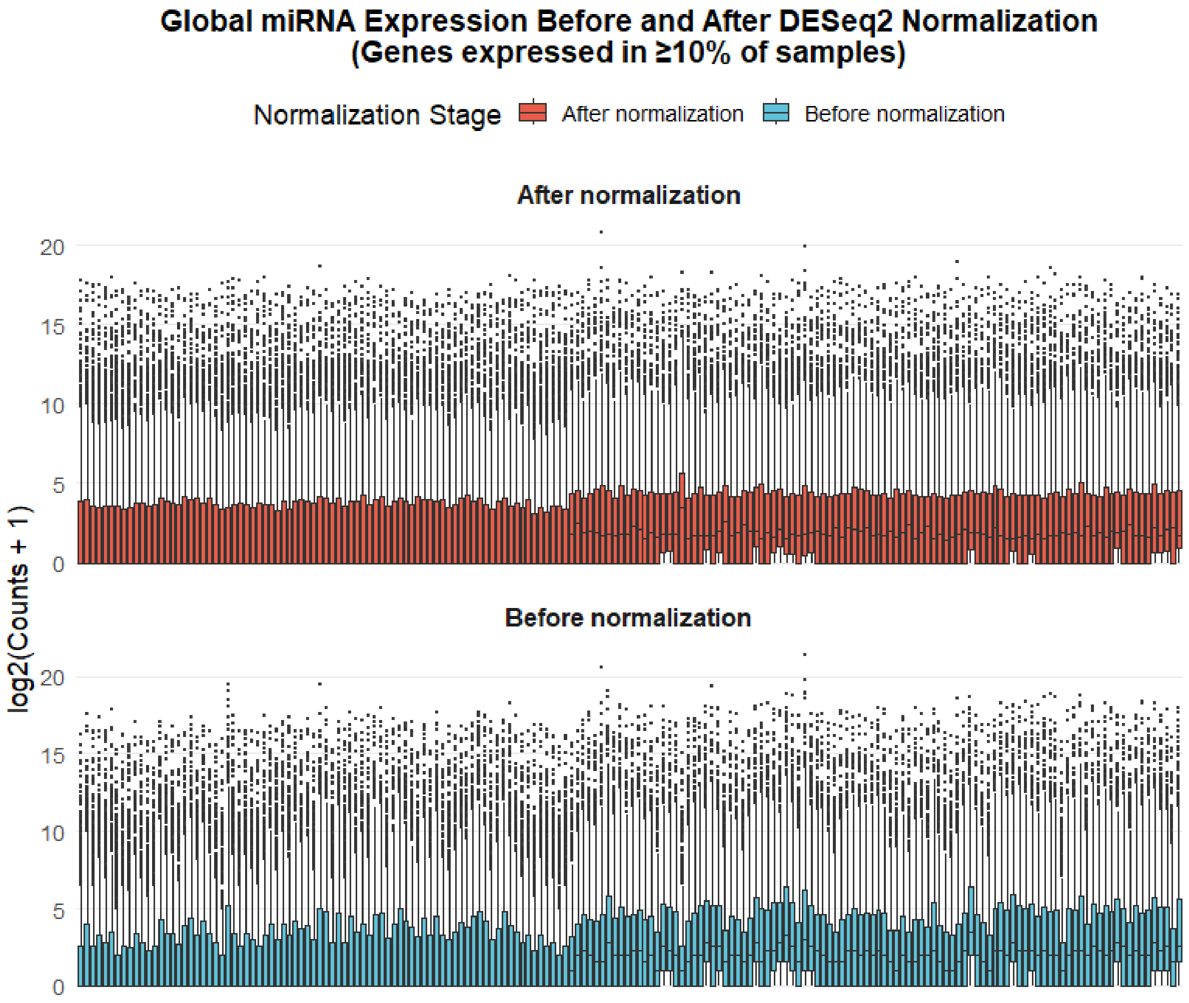

Supplementary Figure S3. Differential expression of mTOR pathway genes in high-grade serous ovarian cancer.

Diverging bar plot showing log2 fold changes (log2FC) of mTOR pathway genes in high-grade serous ovarian cancer (HGSOC) compared with normal ovarian tissue. Bars extending to the right indicate upregulated genes, whereas bars extending to the left indicate downregulated genes. Colour intensity represents the magnitude of the expression change.

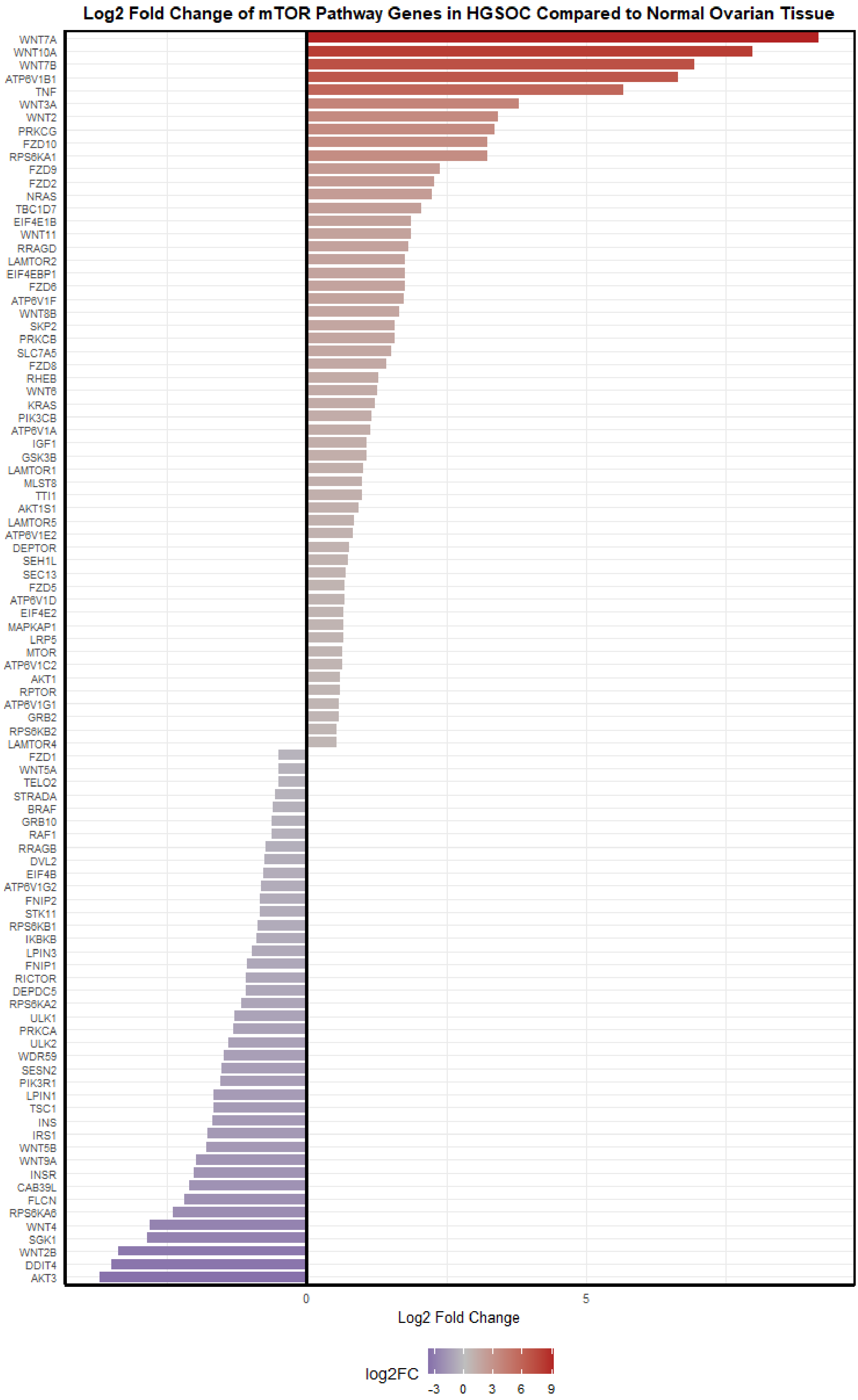

Log2 Fold Change of mTOR Pathway Genes in HGSOC Compared to Normal Ovarian Tissue
WNT7A
WNT10A
WNT7B
ATP6V1B1
TNF
WNT3A
WNT2
PRKCG
FZD10
RPS6KA1
FZD9
FZD2
NRAS
TBC1D7
EIF4E1B
WNT11
RRAGD
LAMTOR2
EIF4EBP1
FZD6
ATP6V1F
WNT8B
SKP2
PRKCB
SLC7A5
FZD8
RHEB
WNT6
KRAS
PIK3CB
ATP6V1A
IGF1
GSK3B
LAMTOR1
MLST8
TTI1
AKT1S1
LAMTOR5
ATP6V1E2
DEPTOR
SEH1L
SEC13
FZD5
ATP6V1D
EIF4E2
MAPKAP1
LRP5
MTOR
ATP6V1C2
AKT1
RPTOR
ATP6V1G1
GRB2
RPS6KB2
LAMTOR4
FZD1
WNT5A
TELO2
STRADA
BRAF
GRB10
RAF1
RRAGB
DVL2
EIF4B
ATP6V1G2
FNIP2
STK11
RPS6KB1
IKBKB
LPIN3
FNIP1
RICTOR
DEPDC5
RPS6KA2
ULK1
PRKCA
ULK2
WDR59
SESN2
PIK3R1
LPIN1
TSC1
INS
IRS1
WNT5B
WNT9A
INSR
CAB39L
FLCN
RPS6KA6
WNT4
SGK1
WNT2B
DDIT4
AKT3
0
5
Log2 Fold Change
log2FC
-3
0
3
6
9

Supplementary Figure S4. Kaplan–Meier survival analysis of six hub genes in high-grade serous ovarian cancer.

Kaplan–Meier curves show overall survival (OS) stratified by high and low expression (median split) of FNIP1, WNT9A, RICTOR, TSC1, INSR, and FNIP2 in patients with high-grade serous ovarian cancer. Shaded areas represent 95% confidence intervals. Log-rank p-values are indicated on each panel, and hazard ratios (HR) with 95% confidence intervals were estimated using Cox proportional hazards models. High FNIP1 expression was significantly associated with poorer overall survival, whereas the remaining hub genes showed no statistically significant association with OS.

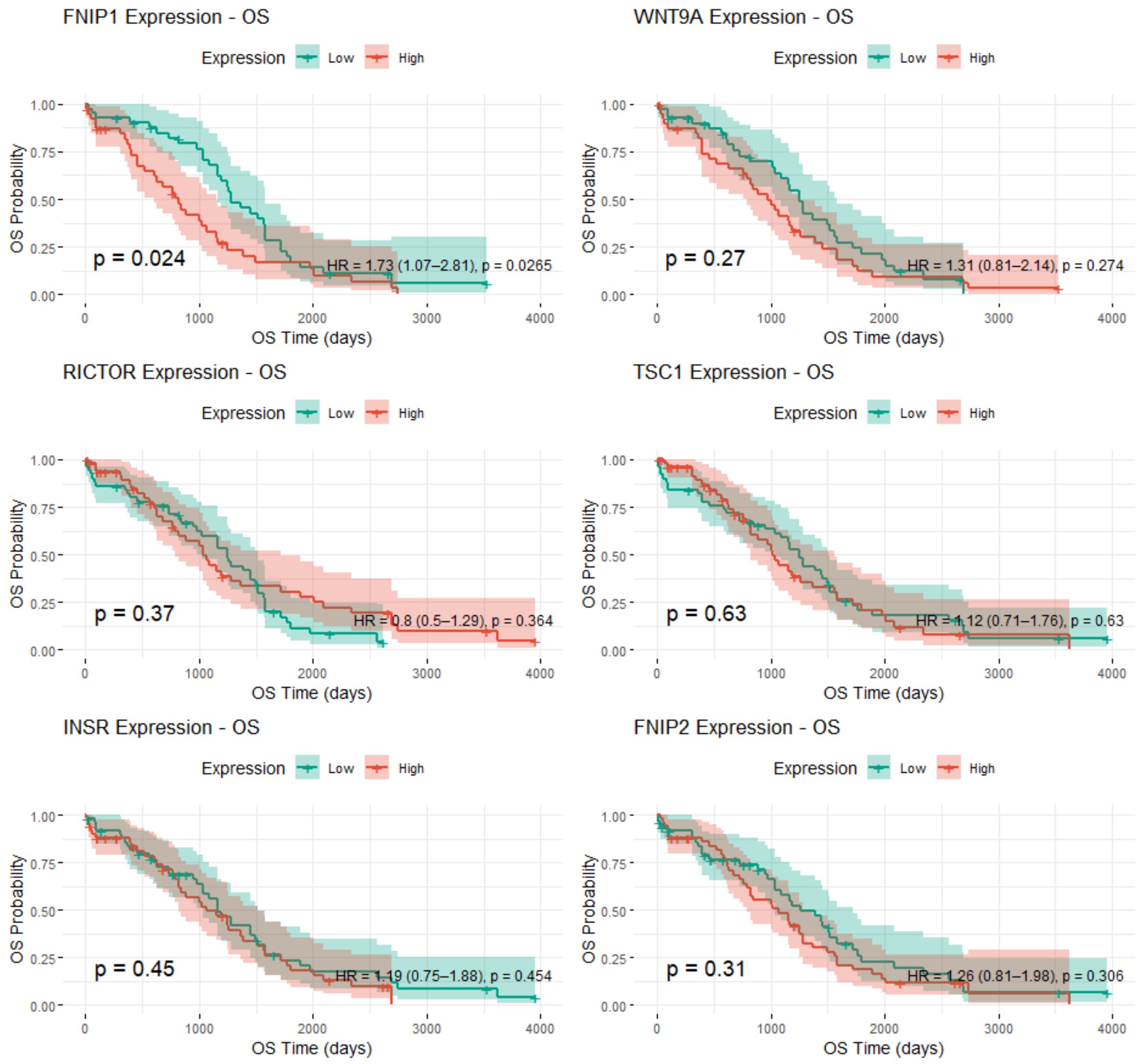

All Supplementary tables are submitted in.xlsx format

Supplementary Table S1. Sample identifiers and source databases of the study cohort.

Supplementary Table S2. miRNA differential expression analysis.

Supplementary Table S3. Predicted miRNA targets, validation status, and mTOR-specific cancer-related miRNA–mRNA interactions, degree centrality

Supplementary Table S4. mRNA differential expression analysis.
